# Validity and Reliability of an Instrumented Treadmill with an Accelerometry System for Assessment of Spatio-Temporal Parameters and Impact Transmission

**DOI:** 10.3390/s21051758

**Published:** 2021-03-04

**Authors:** Alberto Encarnación-Martínez, Pedro Pérez-Soriano, Roberto Sanchis-Sanchis, Antonio García-Gallart, Rafael Berenguer-Vidal

**Affiliations:** 1 Research Group in Sports Biomechanics (GIBD), Department of Physical Education and Sports, University of Valencia, 46010 Valencia, Spain; pedro.perez-soriano@uv.es (P.P.-S.); roberto.sanchis@uv.es (R.S.-S.); 2 Physical Education and Sport, University of Alicante, 03690 San Vicente del Raspeig, Spain; 3 The Civil Guard, Secretary of State for Security, Ministry of the Interior, 28010 Madrid, Spain; garciagallart@gmail.com; 4 Grupo de Investigación en Telecomunicaciones Avanzadas (GRITA), Catholic University of Murcia, 30107 Guadalupe, Spain; rberenguer@ucam.edu

**Keywords:** impact acceleration, spatio-temporal, instrumented treadmill, running, retraining

## Abstract

Running retraining programs focused on concurrent feedback of acceleration impacts have been demonstrated to be a good strategy to reduce running-related injuries (RRI), as well as to improve running economy and reduce acceleration impacts and injury running incidence. Traditionally, impacts have been registered by mean of accelerometers attached directly to the athletes, which is inaccessible to the entire population, because it requires laboratory conditions. This study investigated the validity and reliability of a new device integrated directly into the treadmill, compared to a traditional acceleration impact system. Thirty healthy athletes with no history of RRI were tested on two separate days over the instrumented treadmill (AccTrea) and simultaneously with an acceleration impact system attached to the participant (AccAthl). AccTrea was demonstrated to be a valid and reliable tool for measuring spatio-temporal parameters like step length (validity intraclass correlation coefficient (ICC) = 0.94; reliability ICC = 0.92), step time (validity ICC = 0.95; reliability ICC = 0.96), and step frequency (validity ICC = 0.95; reliability ICC = 0.96) during running. Peak acceleration impact variables showed a high reliability for the left (reliability ICC = 0.88) and right leg (reliability ICC = 0.85), and peak impact asymmetry showed a modest validity (ICC = 0.55). These results indicated that the AccTrea system is a valid and reliable way to assess spatio-temporal variables, and a reliable tool for measuring acceleration impacts during running.

## 1. Introduction

Running is one of the most popular recreational activities [1,2,3]. Its success may be because it is an aerobic activity that improves health and longevity, prevents diseases, and is very effective for getting fit [1,2,3]. Against the numerous benefits of running, injuries in this activity have a high incidence as almost half of runners are injured every year [1]. The annual incidence of lower-limbs injuries ranges from 19.4% to 79.3% [1], or even 92.4% [2] in long-distance runners. Most injuries are caused by the overuse of certain structures, [1,2,4] and the knee is the most common place of injury [2,4], ranging from 7.2% to 50% [2]. Thus, injuries can lead to a temporary or permanent interruption of exercise and even inability to work, leading to the need for medical treatment, where direct costs may exceed 1300 € [5].

Scientifically related to running injuries [6,7,8], an impact is generated with each foot contact with the floor that produces stress up to 1.5 to 2.5 times the body weight [8], and it is transmitted and absorbed by the whole body [9,10,11]. These impacts are attenuated internally by passive structures such as bones, cartilage, and ligaments, and by active movements such as joint angular displacements and eccentric muscle actions, in addition to external components such as footwear or surfaces [11]. The impacts during running have been broadly studied, and accelerometry is the technique most used to register this mechanical stress in sports activities [6,7,8,11]. This technique is based on the placement of low-mass accelerometers (uniaxial or triaxial), mostly on the tibia and front of the head to register in “g” or gravities (1 g = 9.8 m/s^2^) the acceleration/deceleration of body segments to calculate the magnitude and attenuation of impact [8,9,10,11]. It has been found that after a prolonged running fatigue protocol, while tibial accelerations increase [6,7,8], head accelerations remain stable [7,8,11], which means that impact absorption also increases [6,8,12]. Therefore, it is necessary to adopt measures to reduce these stress levels on the musculoskeletal system during running and their negative effects [4].

The running surface, as an external component, plays a major role in impact attenuation [11,13]. Although overground running is the surface preferred for recreational runners [14], running on a treadmill is a very popular activity in gyms, in therapeutic activities, rehabilitation, training, or athletic performance testing [13,15]. It has been shown that running on a treadmill can modify running biomechanics compared with overground running [16]. These kinematic modifications during treadmill running favors a running technique characterized by a higher level of security [17] as the magnitude of the impact is lower [13,17] and the risk of stress injuries is lower on treadmills in comparison with overground running [18]. Around 5000 impacts can occur during a typical 30 min running practice [8]. Thus, an excessively high impact level, due to a poor running technique or a reduction in attenuation ability as the fatigue progresses, has been related to an increased risk of injury [6,7,8].

Despite the potential benefits associated with running on a treadmill, the spatial and sensory constraints imposed by treadmills alter temporal and neuromuscular control in comparison with the overground condition [19]. Nevertheless, some research focused on analyzing the effects of biofeedback or auditory or visual information on some modifiable factors, such as running technique, that could reduce the severity of impacts [20,21,22,23]. These authors showed that by providing visual [21] or auditory [22] information through a screen about the impact levels received during treadmill running, athletes were able to make small modifications in their running technique autonomously to lower the impact peak [21,22], and their running technique became more efficient or economical [20]. Therefore, the implementation of biofeedback is an effective measure to reduce impacts and improve running economy [20,21,22].

It is important to highlight that all the studies that analyze impacts during running using accelerometry place the sensors directly on the athletes’ body, and the biofeedback system is used as an external element to the instruments used to carry out the activity. Similarly, to analyze spatio-temporal variables during running, other systems based on contact platforms [24] or optoelectronic technology [25] have been previously used. However, these systems allow just a limited number of strides or require expensive technology, making them inaccessible to the general population.

Some research works have used instrumented treadmills with force-plates or pressure sensors that allow measurement of the pressure produced by the runner on the lower board of the treadmill [26,27]. Force-plates present interesting advantages in motion and gait analysis [28], although the substantial cost of this instrumentation reduces the possibility of its use outside the laboratory on a large scale. On the other hand, despite the numerous advantages of using accelerometers for impact analysis described above, as far as we know to date [29,30,31], there are no treadmills that integrate acceleration sensors into their own system.

Accelerometers are today a proven and low-cost technology used for displacement estimation [32] in a wide range of applications, such as electrohydraulic systems [33], architecture, civil engineering [34,35], seismology [36], or even astronomy [37]. In all these applications, the accelerometers are rigidly attached to the element whose displacement is to be monitored, and with an analysis of the accelerometry signals, the motion and other parameters of interest can be estimated. For this reason, we proposed the placement of accelerometry sensors directly on a treadmill, which will allow us to similarly estimate the movement on the treadmill and thus analyze the movement of the runner when using the treadmill.

Thus, our aims were: (a) To implement and validate an accelerometry system, placed directly in the treadmill and integrated into the software; (b) to compare the impact and space–time data during running obtained from the accelerometry system integrated in the treadmill with the data extracted from the accelerometry system placed directly on the athlete’s body. We hypothesize that: (a) The accelerometry system integrated in the treadmill is a valid and reliable tool for measuring impacts and space–time parameters during running; (b) the accelerometry system integrated in the treadmill offers similar data to those provided by an accelerometry system placed directly on the athlete’s body.

## 2. Materials and Methods

### 2.1. Participants

This study was approved by the institution’s Human Research Ethics Committee (registry number: 6775). Thirty recreational athletes, ten women and twenty men, were recruited from local Athletics recreational teams, from March to April 2019, and were tested twice. Both tests were completed within 2 weeks and at least 24 h apart. Inclusion criteria were: To be physically active (to run a minimum of twice a week in the last year, do 2 h and 30 min a week of moderate-intensity, or 1 h and 15 min a week of vigorous-intensity aerobic physical activity), to have no history of lower body injuries within the last six months, to not be taking medication that hinders stability during the running, and to not suffer musculoskeletal disorders, heart failure, or neurological disorders that could affect normal locomotion. Athletes were excluded if they have had significant illness, injury, or surgery within the previous six months, and if they were overweight or obese (BMI < 24.9 kg/m^2^). All participants provided informed consent before their inclusion in the study. The baseline characteristics are shown in Table 1.

### 2.2. Experimental Setups

Acceleration impact data during running were recorded using a wireless triaxial accelerometry system (AcelSystem, Blautic, Spain; dimensions: 40 mm × 22 mm × 12 mm) adjusted to the athletes (AccAthl), at a sampling ratio of 415 Hz, a measuring range of up to ±16 g, and a total mass of 2.5 g. Simultaneously, a system consisting of a set of four triaxial MPU-9250 accelerometry sensors (TDK InvenSense, San José, CA, USA) embedded in the treadmill (AccTrea) was used. These four accelerometers were set at a sampling frequency of 250 Hz and with a range up to ±8 g, appropriate for the expected measurement values [29,30,31].

**Figure 1:**
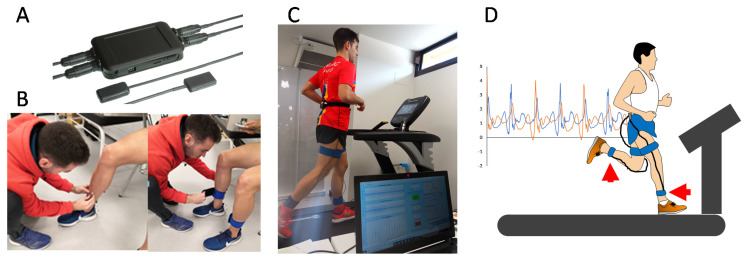
Graphical schematic of the body-worn accelerometer fixed to athletes: (**A**) Accelerometry system (AcelSystem, Blautic); (**B**) accelerometer fixation on distal and anteromedial portion of each tibia; (**C**) final experimental setup; and (**D**) graphical representation of the accelerometer signal and the experimental setup.

For every participant, a lightweight triaxial accelerometer was placed on the distal and anteromedial portion of each tibia with the vertical axis of each accelerometer aligned to be parallel to the long axis of the shank [38], as the location of the tibial accelerometer does influence the acceleration signal [38]. The skin was previously prepared and the accelerometers were adjusted with elastic belts as recommended by Encarnación-Martínez, García-Gallart, Gallardo, Sánchez-Sáez, and Sánchez-Sánchez [9] (Figure 1). The treadmill accelerometry system was encased inside the treadmill (EVOT1, Bodytone International Sports, Murcia, Spain), and comprised three parts: A group of triple-axis Micro Electro-Mechanical System (MEMS) accelerometers, a data acquisition unit, and a processing unit. Appendix A details the operation and connection between these constituent elements of AccTrea. Both AccAthl and AccTrea systems were triggered simultaneously to collect the impact acceleration data.

Participants performed two running tests on different days. The first session intended to assess the validity of the accelerometry system implemented in the treadmill (AccTrea) versus an accelerometry system adjusted to the athletes (AccAthl), and the second session intended to test the reliability. Both running accelerations’ measurement sessions were undertaken in the biomechanics lab at the same environmental conditions and at similar times of the day. All participants used the heel–toe running style and wore their own running shoes (the same for all two tests). After the informed consent, participants performed a free 5 min warm-up until they were familiar with the testing treadmill condition [39]. Next, the participants were instrumented with the accelerometers and the running tests were performed. They ran for 5 min at 10 km/h and 0% slope in order not to affect the parameters evaluated [40], and acceleration impacts and spatio-temporal parameters were collected by the AccTrea and the AccAthl systems in two sets of 10 s during the last minute taken in each measurement session. Rate of Perceived Exertion (RPE) [41] was also registered after the warm-up and after each of the running test.

The vertical component (*z*-coordinate) of the accelerometry signals has been proven to be most important for the assessment of acceleration impacts and injury stroke incidence [42]. Therefore, in both AccAthl and AccTrea, the vertical component of all accelerometers was gathered for analysis.

Data from the AccAthl system were analyzed using the Matlab program (MathWorks, MA, USA), custom-made. The accelerometers were previously calibrated by the manufacturer. The acceleration signal from each of the sensors was first filtered (Butterworth, second-order, low-pass, cut-off frequency = 50 Hz) [43]. The signal was then segmented by calculating the signal period (using the autocorrelation) and locating the points of interest (maximum, minimum, etc.) for each step. The positive peak tibial acceleration was measured for each leg in g (1 g = 9.82 m/s^2^), as well as the asymmetry between the legs, calculated as the relative difference between both peaks (right leg impact minus left leg impact) expressed as a percentage (%).

On the other hand, as detailed in Appendix A, AccTrea incorporated four MPU-9250 sensors. According to the manufacturer’s specifications, these devices included a motion processing unit with low-pass filters and an EEPROM for on-chip factory calibration of the sensor. Thus, factory-trimmed scale factors eliminated the need for external active components and end-user calibration. Nevertheless, a calibration routine was performed at sensor initialization on the data acquisition unit to offset the bias of gravity [44].

**Figure A1:**
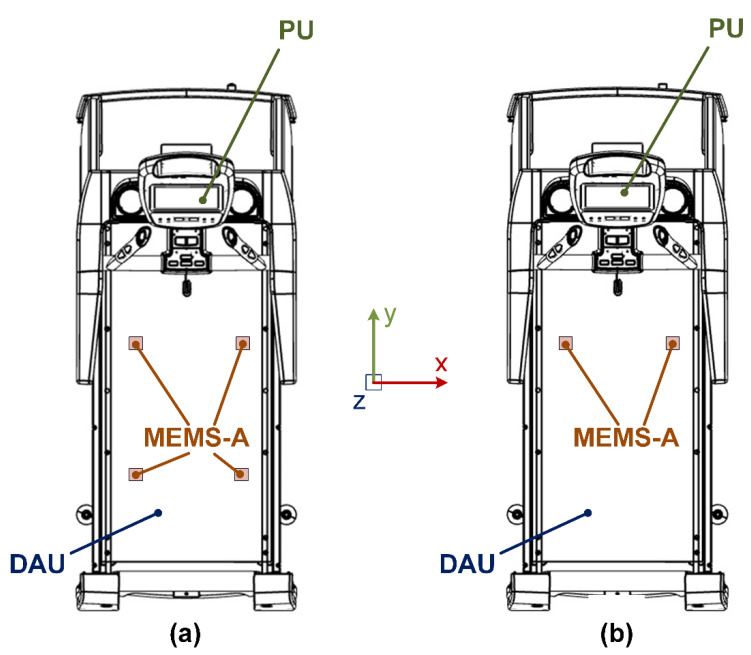
Location of accelerometers (MEMS-A), data acquisition unit (DAU), and processing unit (PU) on the treadmill (AccTrea): (**a**) Four-accelerometer approach; (**b**) two-accelerometer approach.

The AccTrea system allowed us to measure the acceleration of the table of the treadmill due to the runner impacts at each sensor position (see Figure A1). The difference in amplitude and phase between the signals from the different sensors made it possible to automatically detect the accelerations produced by each leg. Then, like AccAhtl, the asymmetry between the legs was also calculated from the signals of these sensors.

Finally, the accelerometry data from both AccAhtl and AccTrea approaches were analyzed using Matlab (R2015a with Signal Processing Toolbox, MathWorks Inc., Natick, MA, USA), providing a set of spatio-temporal parameters such as step time (ms), step length (m), and step frequency (spm), that allowed a comparison of the two approaches. Appendix B details the algorithms used for calculating these parameters.

### 2.3. Statistics

Prior to the validity and reliability tests, a chi-square test was performed to determine whether there were differences between males and females. The agreement between the two systems was reviewed by a Bland–Altman plot for each of the variables analyzed. The differences between the two systems (AccTrea–AccAthl) in each variable were plotted against the mean results [45]. Reliability was contrasted by means of a two-way, random-effects, single-measure (median of the two trials) intraclass correlation coefficients (ICC(2,1)) model. In conjunction with the ICC values, standard error of measurement (SEM) and minimum detectable change (MDC) values were calculated to assess the concurrent validity between the AccTrea and the AccAthl, as well as the within-device test–retest reliability and measurement error over the two testing sessions for all outcome measures [46]. Point estimates of the ICCs were interpreted as follows: Excellent (0.75–1), modest (0.4–0.74), or poor (0–0.39) [47]. All statistical analyses were conducted using the Statistical Package for the Social Sciences (SPSS Inc. Version 26.0, Chicago, IL, U.S.A.). The MDC, which is otherwise known as the reliable change index score, was calculated using the equations reported previously by Jacobson and Truax [48]. It is expressed as the percentage test–retest change in impact acceleration or spatio-temporal parameter required to find a significant difference at an alpha level of 0.05 based on the Day 1 mean value.

## 3. Results

### 3.1. Gender Differences

The results of the chi-square test showed no statistically significant differences (mean bilateral asymptotic significance 0.411) regarding gender for any of the variables analyzed. Therefore, during this study, all subsequent statistical analyses were conducted jointly, including men and women, as a single sample for each of the groups.

### 3.2. Perceived Exertion

Regarding the perceived exertion, no differences were found between sessions for any of the study groups (Table 2).

### 3.3. Bland–Altman Plots

**Figure 2:**
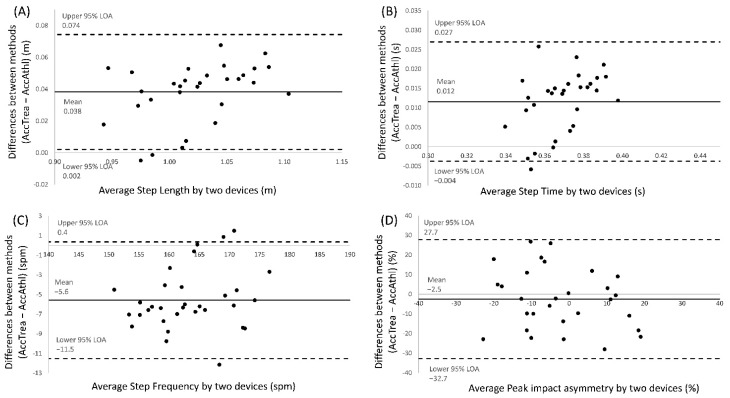
Bland–Altman plots representing comparisons between the AccTrea system and the AccAthl system for four of the variables analyzed: (**A**) Step length; (**B**) step time (duration); (**C**) step frequency; and (**D**) peak acceleration impact asymmetry. The mean line represents the mean difference between the devices, with the upper and lower dashed lines representing the 95% limits of agreement (LOAs).

All participants successfully completed the two days’ sessions. The Bland–Altman plots for the step length, step time, step frequency, and peak acceleration impact asymmetry are provided in Figure 2. There was a small relationship between the difference and the mean for all the spatio-temporal variables. Specifically, step length and time were slightly lower in the AccAthl system compared to AccTrea, and as a result, the step frequency variable was higher in the AccAthl system. The acceleration impact asymmetry did not show any obvious relationship between systems.

### 3.4. Validity and Reliability

The results for the step length, step time, step frequency, left leg peak acceleration impact, right leg peak acceleration impact, and peak acceleration impact asymmetry variables are provided in Table 3. The step length and step time were lower in the AccAthl system compared with AccTrea. Step frequency, left leg peak acceleration impact, and right leg peak acceleration impact variables showed a bias toward higher values in the tests performed on the AccAthl. Inconsistent results were found for peak acceleration impact asymmetry variables.

In general, both systems showed excellent test–retest reliability (Table 3), with only the peak acceleration impact asymmetry values’ performance on the AccTrea (ICC = 0.36) failing to reach an ICC value of 0.75, considered as an excellent value. Concurrent validity was shown to be consistently excellent across spaciotemporal variables and testing sessions (ICC = 0.94–0.98), but not in acceleration impact variables for every testing session (ICC = −0.01–0.55). The SEM for the spaciotemporal variables ranged from 0.92 to 1.31% in the AccTrea system, and from 1.19 to 1.29% in the AccAthl system. For impact variables, the SEM ranged from 10.1 to 358% in the AccTrea system and from 12.25 to 297% in the AccAthl system.

The MDC in all variables ranged from 0.04 to 27.8% for the AccTrea system and from 0.04 to 20.2% for the AccAthl system. The MDCs were reasonably high for both devices only in the peak acceleration impact asymmetry variable (27.8% at AccTrea and 20.2% at AccAthl). With respect to the other variables (spaciotemporal and impacts), the MDCs were lower for both systems, with the AccAthl MDC values higher than the AccTrea values in all values.

## 4. Discussion

Validity and reliability of spatio-temporal and impact transmission variables during running are important in biomechanical analysis under laboratory conditions.

Treadmills are becoming popular between recreational runners [15]. Oxygen uptake, heartrate, and perceived effort are similar between submaximal treadmill and overground running [15]. However, running on a treadmill provides greater control over environmental variables such as temperature, wind speed, or relative humidity [15]. Treadmills also offer control over running velocity and surface gradient [15], and generate changes in biomechanics parameters like step length, contact time, and stride frequency compared with overground running [17].

These kinematics modifications favor the reduction in impact acceleration magnitude [13,17], axial compression strains in tibia [18], and plantar load [13,49] in comparison with overground running. It causes runners to adopt a safer running style [17].

In addition, running retraining programs, focused on reducing the severity of impacts that are related to running injuries, have demonstrated good results by means of introducing biofeedback systems (auditory or visual information) during training sessions on the treadmill. Previous studies have shown that runners were able to reduce impacts and improve running economy thanks to the concurrent information about the severity of the impacts received from accelerometers placed directly on their body [20,21,22].

The control of environmental and performance factors, along with the kinematic modifications offered by the treadmills, can make it a safer activity than overground running. Introducing auditory or visual biofeedback information from the treadmill could allow the control of impact acceleration and make that system accessible for all types of runners, both professional and recreational.

Our results partially confirmed the hypothesis raised in the study, that the AccTrea system integrated in the treadmill is a valid and reliable tool for measuring spatio-temporal parameters like step length (validity ICC 95%CI = 0.87/0.97; reliability ICC 95%CI = 0.82/0.96), step time (validity ICC 95%CI = 0.87/0.97; reliability ICC 95%CI = 0.90/0.98), and step frequency (validity ICC 95%CI = 0.90/0.97; reliability ICC 95%CI = 0.91/0.98) during running on a treadmill compared to the AccAthl system under the same speed condition. Nevertheless, peak acceleration impact variables measured during running showed a high reliability for the left leg (reliability ICC 95%CI = 0.75/0.94) and right leg (reliability ICC95%CI = 0.69/0.93), but not a high validity (Table 2). On the other hand, peak acceleration impact asymmetry showed a modest validity (ICC = 0.55) but a poor reliability (Table 2).

Prior to our study, other systems that measure spatio-temporal variables during running have been validated. These systems were initially based on contact platforms [24], but they allowed the analysis of just a limited number of strides, in addition to the possibility of altering the running gait. Other systems based on optoelectronics technology were also validated [25] to measure the spatio-temporal variables without altering the natural running pattern [50], but the drawback of these systems was that they need to install different extremely sensitive instruments whose technology is relatively expensive compared to the technology of the AccTrea system, analyzed in the present study.

The spatio-temporal variables analyzed in our study have shown intraclass correlation coefficients (ICC > 0.946) close to those obtained by Ogueta-Alday, Morante, Rodríguez-Marroyo, and García-López [50] when they validated a new method to measure contact time and flight time during treadmill running (SportJump System Pro, V2.0., León, Spain) (ICC > 0.993). It should be noted that in the present study, other variables have been analyzed than those evaluated in the SportJump System Pro, as the objective of the AccTrea system was to provide concurrent feedback to runners in order to modify step length, frequency, and time to improve their running economy.

The excellent validity and reliability results for the spatio-temporal variables, together with the technology used, make the AccTrea system a low-cost and high-reliability system, nonexistent until now.

The results of peak acceleration impact asymmetries are considered modest (ICC = 0.55) for validity between systems and poor (ICC = 0.36) for the within-device test–retest reliability for the AccTrea system. Both acceleration impact peaks of the left leg (ICC = 0.88) and right leg (ICC = 0.85) obtained a high degree of reliability of the AccTrea system between days, which was not the case for validity between systems (AccTrea and AccAthl), considered as poor (ICC = 0.01).

Symmetry/asymmetry in running is very difficult to keep within the same values between different sessions as there are many factors that affect running technique [51]. It is a personal technical factor subject to the variability in the dynamic complex systems, an aspect that makes the standardization of the results difficult [51]. The values obtained in the present study were relatively low (little asymmetry on impacts between legs), which could justify the poor reliability results of the system between days.

The poor validity results between systems obtained in the acceleration impact peak variables could be related to the fact that the AccTrea system, compared to the AccAthl, presents elements that could favor the reduction or loss of acceleration and impact dissipation. These elements could be classified as elements typical of the runner, such as the cushioning of the shoes [52]; or elements of the system itself (AccTrea), such as the treadmill, the table, or the protection of the accelerometers, that avoid their displacement and make them register lower values [53].

The Bland–Altman plots demonstrated low mean differences and wide limits of agreement (LoAs) of 95%, except for the step frequency variable, with a mean difference between systems of ±5 ppm. These differences could be explained because step time is also slightly lower in the AccAthl system, possibly associated with acceleration losses of the system previously mentioned.

Regarding the system, there are currently no studies with which the results obtained from the acceleration impact variables can be compared. There are also no systems on the market that can directly or indirectly measure the acceleration impact variables without instrumenting the athlete and with the technology used inserted directly into the treadmill.

Previous studies that have analyzed the effect of immediate biofeedback, via auditory [22] or visual [21], during running have shown that the maximum impact peak was significantly reduced [21,22], improving running economy [20]. Recent studies have shown that the effects of an intervention, applying instant feedback, can last up to a year after the intervention, notably improving the reduction of impacts and reducing the percentage of injured athletes [54].

However, all these biofeedback systems used in previous studies have required athletes to be instrumented with expensive systems and under laboratory conditions, making the use of this type of system impractical on a recurring basis by the general population. The implementation of a biofeedback system, such as the one analyzed in this study, represents a step forward to make impact reductions and running economy improvements accessible to the entire population [23] thanks to its low cost and the unneeded instrumentation of athletes.

The results of this study determined that the system has moderate validity for the scientific measurement of the acceleration impact variables (ICC = 0.55), but it can also be transferred to the sports world, being a valid approximation like the contributions that already exist in the market in the measurement of other variables.

## 5. Conclusions

AccTrea is a reliable and valid tool for athletes to be informed, in a concurrent way, of their biomechanical responses in relation to spatio-temporal variables (step length, step time, and step frequency) during running on an instrumented treadmill. On the other hand, the limitations found in the placement of the accelerometers under the treadmill, which in turn, are great advantages of the system by not having to instrument the athletes, make AccTrea a reliable system measuring running impacts. While peak acceleration impact asymmetry variables presented a modest validity between systems. As a noninvasive biofeedback system for running biomechanical response, AccTrea demonstrates potential as a commercial system of easy access to the general population, with high reliability in spatio-temporal variables and peak acceleration impacts. MEMS sensor technology coupled with a data acquisition unit and a processing unit connected with the treadmill can provide accurate and objective data to improve running mechanics or to allow personal trainers to select running exercises in order to change running mechanics.

## 6. Patents

European patent application with reference EP3735900A1 and entitled “Treadmill for sport training” in May 2019.

U.S. patent application with reference US20200353309A1 and entitled “Ergometric treadmill for sport training” in May 2019.

Chinese patent application with reference CN111905333A and entitled “Force measuring running machine for sports training” in May 2019.

## Figures and Tables

**Table 1:** Baseline characteristics of the thirty participants, values are means ± SD.

Characteristics (M ± SD)	Female (n = 10)	Male (n = 20)
Age, y	24.4 ± 6.1	27.2 ± 7.5
Weight, kg	55.8 ± 4.0	73.3 ± 8.0
Height, cm	161.3 ± 4.3	175.6 ± 5.1
BMI, kg/m^2^	21.4 ± 1.3	23.7 ± 2.3

M = mean, SD = standard deviation, BMI: Body mass index.

**Table 2:** Rate of perceived exertion (RPE) differences between sessions at warm-up and the running test.

	Day 1	Day 2	*p* Value ^1^
Warm-up (M ± SD)	9.0 ± 1.9	8.8 ± 2.0	0.68
Running test (M ± SD)	9.8 ± 1.6	9.5 ± 1.8	0.46

^1^ RPE differences between days (*t*-test).

**Table 3:** Validity and reliability of an instrumented treadmill with an accelerometry system for assessment of spatio-temporal parameters and impact transmission.

	AccTrea	AccAthl	Mean Diff (95%CI)	ICC (95%CI)
**Step Length (m)**				
Day 1 (M ± SD)	1.04 ± 0.05	1.01 ± 0.04	0.04 (0.03/0.05)	0.94 (0.87/0.97)
Day 2 (M ± SD)	1.03 ± 0.04	1.00 ± 0.05	0.04 (0.03/0.05)	0.95 (0.89/0.98)
Mean Diff (95%CI)	0.002 (−0.005/0.010)	0.011 (−0.002/0.023)		
ICC (95%CI)	0.92 (0.82/0.96)	0.88 (0.73/0.95)		
SEM (% SEM)	0.01 (1.31)	0.01 (1.29)		
MDC (%)	0.04	0.04		
**Step Time (ms)**				
Day 1 (M ± SD)	374.8 ± 16.8	363.1 ± 13.9	12.5 (9.7/15.3)	0.94 (0.87/0.97)
Day 2 (M ± SD)	371.8 ± 15.2	359.7 ± 17.1	13.0 (10.1/15.9)	0.95 (0.89/0.98)
Mean Diff (95%CI)	0.88 (−1.75/3.51)	3.85 (−0.60/8.29)		
ICC (95%CI)	0.96 (0.90/0.98)	0.89 (0.73/0.95)		
SEM (% SEM)	3.55 (0.95)	4.7 (1.29)		
MDC (%)	9.85	13.03		
**Step Frequency (spm)**				
Day 1 (M ± SD)	160.5 ± 7.2	166.1 ± 6.6	−5.59 (−6.7/−4.5)	0.95 (0.90/0.97)
Day 2 (M ± SD)	161.3 ± 6.7	167.1 ± 8.0	−5.94 (−7.2/−4.7)	0.95 (0.89/0.98)
Mean Diff (95%CI)	−0.17 (−1.24/0.89)	−0.80 (−2.9/1.3)		
ICC (95%CI)	0.96 (0.91/0.98)	0.91 (0.82/0.93)		
SEM (% SEM)	1.48 (0.92)	1.97 (1.19)		
MDC (%)	4.12	5.47		
**Left Leg Peak Impact (g)**				
Day 1 (M ± SD)	0.72 ± 0.21	3.76 ± 1.37	−3.04 (−3.53/−2.56)	0.09 (−0.86/0.56)
Day 2 (M ± SD)	0.72 ± 0.22	3.93 ± 1.30	−3.21 (−3.69/−2.73)	0.08 (−0.93/0.56)
Mean Diff (95%CI)	0.001 (−0.052/0.053)	−0.181 (−0.514/0.152)		
ICC (95%CI)	0.88 (0.75/0.94)	0.88 (0.74/0.94)		
SEM (% SEM)	0.07 (10.05)	0.48 (12.25)		
MDC (%)	0.20	1.34		
**Right Leg Peak Impact (g)**				
Day 1 (M ± SD)	0.73 ± 0.20	3.91 ± 1.62	−3.18 (−3.76/−2.59)	0.01 (−1.04/0.52)
Day 2 (M ± SD)	0.76 ± 0.18	3.97 ± 1.71	−3.21 (−3.85/−2.56)	−0.01 (−1.13/0.52)
Mean Diff (95%CI)	−0.03 (−0.08/0.02)	−0.05 (−0.42/0.32)		
ICC (95%CI)	0.85 (0.69/0.93)	0.90 (0.80/0.95)		
SEM (% SEM)	0.08 (10.20)	0.50 (12.64)		
MDC (%)	0.21	1.39		
**Peak Impact Asymmetry (%)**				
Day 1 (M ± SD)	−2.80 ± 12.53	−1.29 ± 14.49	−1.51(15.11/2.67)	0.55 (0.07/0.78)
Day 2 (M ± SD)	−2.75 ± 9.79	2.44 ± 17.94	−6.16 (18.34/3.47)	0.28 (−0.55/0.67)
Mean Diff (95%CI)	0.75 (14.16/2.63)	−3.82 (14.94/2.77)		
ICC (95%CI)	0.36 (−0.37/0.70)	0.75 (0.46/0.88)		
SEM (% SEM)	10.04 (−358.66)	7.28 (297.729)		
MDC (%)	27.82	20.17		

AccTrea: Treadmill system; AccAthl: Athlete system; M: Mean; SD: Standard deviation; CI: Confidence interval; ICC: Intraclass correlation coefficient; Diff: Difference; SEM: Standard error of the measurement; MDC: Minimum detectable change, expressed as a percentage of the Day 1 mean value.

**Figure A2:**
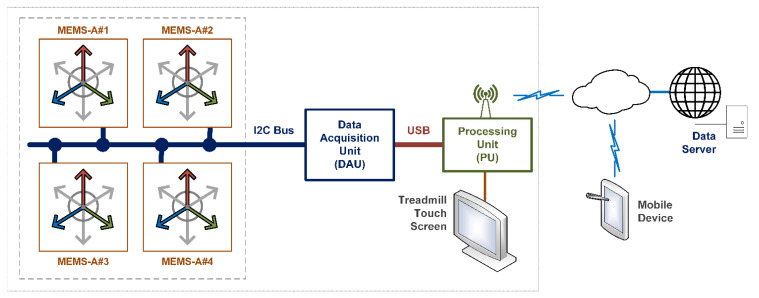
Block diagram of the AccTre system: MEMS-A connected to the DAU via an I^2^C bus; DAU linked to the PU via an USB connection; graphical user interface for biofeedback to the runner on the treadmill touch screen; transmission of processed data to a cloud server and access to the data set through a mobile device.

**Figure A3:**
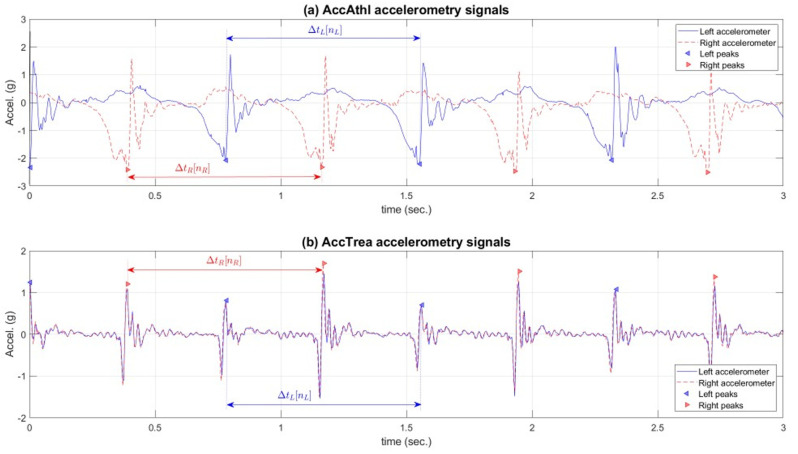
Comparison of the accelerometry signals from the system AccAthl (**a**) and the two-accelerometer approach AccTrea (**b**). The solid and dashed line display the accelerations corresponding to the left and right legs, respectively. The left- and right-pointing triangles mark the acceleration peaks of the left and right legs, respectively.

## Appendix A. Treadmill Accelerometry System (AccTrea)

The proposed measurement system comprises three main parts: A set of triple-axis MEMS accelerometers (MEMS-As), a data acquisition unit (DAU), and a processing unit (PU). All components are encased inside the treadmill. Thus, the system does not require external instrumentation. The MEMS-As send the data set to the DAU, which performs the first stage of filtering and conditioning of the data. This pre-processed data set is then used by the PU to estimate the parameters under interest as detailed in Section 2.

Two different MEMS-A settings can be used in the system. The first approach employs two accelerometers, which are located on the front of the running belt, near the landing zone of the runner. The second approach uses a four-accelerometers setting, where two more accelerometry sensors are placed at the back of the belt. In either approach, all accelerometers are firmly attached to the treadmill board by means of a specifically designed holder in order to maximize the capture of the vibrations produced by the runner. The V120 optical tracking system (Optitrack V120:Trio, NaturalPoint, Inc., Corvallis, OR, USA) has been used to determine the optimal position of the sensors to maximize the measurement of these oscillations. Figure A1 shows the placement of the sensors of the treadmill along with the coordinate system used in the device.

MPU-9250 accelerometry sensors (TDK InvenSense, San José, CA, USA) are used in both settings. These sensors provide digital-output triple-axis accelerometry data with a programmable full-scale range between ±2 g and ±16 g. This feature is particularly interesting as it allows both low-acceleration values, such as when walking on the treadmill, and large values, such as in fast running, to be measured accurately.

MPU-9250 accelerometers use 16-bit analog-to-digital converters (ADCs), which provide enough bit resolution for the subsequent parameter calculation. This allows us to set the scale range to ±8 g, providing a relatively small quantification error and, at the same time, a wide reading range to avoid data clipping. Note that this requirement is important because the acceleration values of the lateral (*x*-axis) and front-rear (*y*-axis) directions are much lower than the vertical one (*z*-axis). Although only the *z*-axis component is analyzed in this work, other accelerometry components may be used in future work. For this reason, the acquisition and storage of all accelerometry components are implemented in this approach.

The MPU-9250 devices include user-programmable digital filters for noise reduction. Although a low-pass filter with a cut-off frequency of 50 Hz is commonly used in many similar applications [38], no in-board filtering has been set in the device for this approach. As shown in Appendix B, the critical information for the calculation of the parameters under analysis involves the time intervals between acceleration peaks. As any low-pass filter smooths these peaks [43], thereby reducing the accuracy of the parameter calculation, the raw data from the sensors are used for further processing and analysis in this approach. Note, however, that prior to any measurement, a calibration is performed on the DAU to compensate for gravity bias [44].

For the connection between the MEMS-A and the DAU, the I^2^C bus is chosen [55]. This protocol allows the transmission of data of all sensors using a single bus, minimizing wiring and system complexity. Each sensor is set-up in a specific address, allowing all accelerometry signals to be transmitted using only two wires.

The choice of sampling frequency of the analog-to-digital converter of the sensors is also a critical issue. A large sampling rate enables a high accuracy in the calculation of the parameters. Nevertheless, it limits the use of multiple sensors with a single I2C bus due to its bandwidth restrictions. A sampling frequency of 250 Hz has been chosen as the compromise value, as it is large enough to obtain sufficient accuracy in the required parameters, while allowing real-time transmission of raw data from up to four sensors.

The data are collected by the DAU, powered by a ATmega2560 microcontroller (Microchip Technology Incorporated, Chandler, AZ, USA). This unit performs the following tasks: (1) Initialization and calibration of the sensors before each training; (2) time synchronization of the signals to be sent as a matrix to the subsequent PU; and (3) monitoring with automatic restart in case of reading or transmission failure.

This data set is transmitted via a USB connection to the PU of the treadmill. This unit is responsible for calculating the parameters listed in Section 2, i.e., positive peak tibial acceleration, asymmetry, step time, step length, and step frequency. In addition, a frequency analysis is carried out, which will allow future work to carry out harmonic analysis, among others. Both Appendix B and patents [29,30,31] detail the process of calculating these parameters. Figure A2 depicts the connection diagram between the MEMS-A, DAU, and PU.

## Appendix B. Procedure for Calculating Spatio-Temporal Parameters

The procedure for calculating these parameters is based on Pérez-Soriano and Encarnación-Martínez [56], although it has been adapted for each of the approaches analyzed in this work. Note that in this study, two accelerometers have been used in both systems. In AccAthl, the sensors are placed on the distal and anteromedial portion of each tibia [38] while in AccTrea, they are located on the front of the running belt [38] (Figure A1).

It is important to note that although both systems measure the acceleration produced by both legs, the way in which both sets of data are collected is completely different. Each AccAthl accelerometer is attached to one of the legs. Thus, the acceleration measured on each leg is clearly recorded on its corresponding accelerometer while the signal caused by the opposite leg is noticeably lower. By contrast, as the accelerometers included in AccTrea are both attached to the rigid board of the treadmill, both accelerometers collect the vibration produced by both legs. Their signals vary only subtly in amplitude depending on the proximity of each sensor to the landing zone of each leg. Nevertheless, this slight difference is sufficient to determine the parameters of interest. Figure A3 illustrates the acceleration levels recorded by each system for the same measurement session.

The first step in the calculation of the parameters is to determine the accelerometry signal peaks for both legs in each approach, including their temporal location (s) and their peak amplitude value (g). To obtain the location $\left\{l_{Ath,L}\left[n_{L}\right],l_{Ath,R}\left[n_{R}\right]\right\}$ and amplitude $\left\{p_{Ath,L}\left[n_{L}\right],p_{Ath,R}\left[n_{R}\right]\right\}$ of the peaks in AccAthl, the maximum negative acceleration values are detected. The variables $n_{L}$ and $n_{R}$ denote the step number for the left and right leg, respectively. These values correspond to the time when the runner lands on each leg. To avoid false-negative detection, constraints on the minimum distance between peaks and on their prominence values are applied.

From the data coming from AccTrea, all the peaks of both accelerometers are detected first. In contrast to the previous approach, the values of the maximum positive acceleration are considered here, as it has been empirically proven that they provide better results for the analysis due to the vibration of the table.

Note that, as shown in Figure A3b, the contribution of both legs is recorded in both accelerometry signals. Therefore, the correspondence of the detected peaks to each leg must be estimated. This is done by averaging the values of the odd and even peaks of each signal and assigning the higher values to the closest sensor. Once this is accomplished, a procedure similar to AccAthl is applied to obtain the location $\left\{l_{Trea,L}\left[n_{L}\right],l_{Trea,R}\left[n_{R}\right]\right\}$ and amplitude $\left\{p_{Trea,L}\left[n_{L}\right],p_{Trea,R}\left[n_{R}\right]\right\}$ of the peaks as depicted in Figure A3b.

From these vectors $\left\{p_{Ath,L}\left[n_{L}\right],p_{Ath,R}\left[n_{R}\right]\right\}$ and $\left\{p_{Trea,L}\left[n_{L}\right],p_{Trea,R}\left[n_{R}\right]\right\}$, statistical values are calculated for the impacts of both legs, as well as for their asymmetry. Table 3 shows these results for both approaches.

The step locations are used to compute the step times using backward differences,

(A1) $$\begin{array}{c} \Delta t_{Athl,L}\left[n_{L}\right]=\frac{10^{3}}{f_{s,Athl}}\left(l_{Ath,L}\left[n_{L}\right]-l_{Ath,L}\left[n_{L}-1\right]\right)\;\mathrm{ms},\\ \Delta t_{Ath,R}\left[n_{R}\right]=\frac{10^{3}}{f_{s,Athl}}\left(l_{Ath,R}\left[n_{R}\right]-l_{Ath,R}\left[n_{R}-1\right]\right)\;\mathrm{ms},\\ \Delta t_{Trea,L}\left[n_{L}\right]=\frac{10^{3}}{f_{s,Trea}}\left(l_{Trea,L}\left[n_{L}\right]-l_{Trea,L}\left[n_{L}-1\right]\right)\;\mathrm{ms},\\ \Delta t_{Trea,R}\left[n_{R}\right]=\frac{10^{3}}{f_{s,Trea_{}}}\left(l_{Trea,R}\left[n_{R}\right]-l_{Trea,R}\left[n_{R}-1\right]\right)\;\mathrm{ms}, \end{array}$$

where $\Delta t_{\left\{⯀,\circ \right\}}\left[n_{\left\{\circ \right\}}\right]$ (ms) denotes the step time for approach {⯀}; leg {∘} and step number, $n_{\left\{\circ \right\}}$ and $l_{\left\{⯀,\circ \right\}}$, respectively, are the peak locations (samples); $f_{s,\left\{⯀\right\}}$ (samples per second) stands for the sampling frequency for each approach; and $\left\{n_{L},n_{R}\right\}$ represents the left and right step numbers, respectively.

The step length is simply calculated by multiplying the step time by the linear speed of the belt, provided by the treadmill electronics,

(A2) $$\Delta l_{\left\{⯀,\circ \right\}}{\left[n_{\left\{\circ \right\}}\right]}_{}=v\cdot 10^{-3}\Delta t_{\left\{⯀,\circ \right\}}\left[n_{\left\{\circ \right\}}\right]\;m$$

where $\Delta l_{\left\{⯀,\circ \right\}}\left[n_{\left\{\circ \right\}}\right]$ (m) denote the step length for the step number $n_{\left\{\circ \right\}}$, $\Delta t_{\left\{⯀,\circ \right\}}\left[n_{\left\{\circ \right\}}\right]$ are the step times calculated in Equation (A1), and v (m/s) represents the linear speed of the belt of the treadmill, which is identical in both approaches.

To determine the step frequency, the number of steps detected by each sensor is counted and divided by the duration of the experiment,

(A3) $$sf_{\left\{⯀,\circ \right\}}=60N_{\left\{⯀,\circ \right\}}/T\;\mathrm{spm}$$

where $sf_{\left\{⯀,\circ \right\}}$ (spm) denotes the step frequency for approach {⯀} and leg {∘}, and T is the duration of the experiment (T=10 s). Once again, the statistical parameters of these variables are shown in Table 3.
